# Research Progress of PPR Proteins in RNA Editing, Stress Response, Plant Growth and Development

**DOI:** 10.3389/fgene.2021.765580

**Published:** 2021-10-18

**Authors:** Tengfei Qin, Pei Zhao, Jialiang Sun, Yuping Zhao, Yaxin Zhang, Qiuyue Yang, Weipeng Wang, Zhuanqing Chen, Tengfei Mai, Yingying Zou, Guoxiang Liu, Wei Hao

**Affiliations:** ^1^ Henan Collaborative Innovation Center of Modern Biological Breeding, Henan Institute of Sciences and Technology, Xinxiang, China; ^2^ State Key Laboratory of Cotton Biology, Institute of Cotton Research, Chinese Academy of Agricultural Sciences, Anyang, China; ^3^ Beijing River and Lake Management Office, Beijing, China; ^4^ Key Laboratory of Tobacco Improvement and Biotechnology, Tobacco Research Institute of Chinese Academy of Agricultural Sciences, Qingdao, China; ^5^ College of Medical Technology, Beihua University, Jilin City, China

**Keywords:** pentatricopeptide repeat, RNA editing, biogenesis, development, mechanism

## Abstract

RNA editing is a posttranscriptional phenomenon that includes gene processing and modification at specific nucleotide sites. RNA editing mainly occurs in the genomes of mitochondria and chloroplasts in higher plants. In recent years, pentatricopeptide repeat (PPR) proteins, which may act as trans-acting factors of RNA editing have been identified, and the study of PPR proteins has become a research focus in molecular biology. The molecular functions of these proteins and their physiological roles throughout plant growth and development are widely studied. In this minireview, we summarize the current knowledge of the PPR family, hoping to provide some theoretical reference for future research and applications.

## Introduction

**FIGURE 1:**
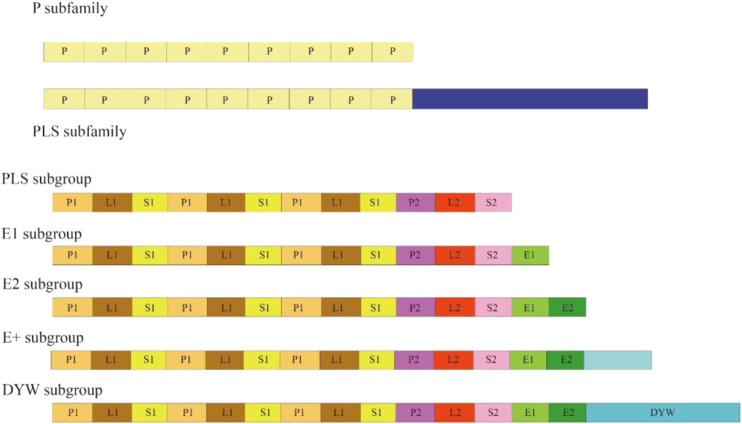
The architecture of different PPR subfamilies and subgroups.

PPR family is one of the largest gene families in higher plants. PPR proteins contain an array of 2–30 tandem repetitions of a degraded unit containing 30–40 amino acid (aa) motifs (Lurin et al., 2004). PPR proteins are classified into two subfamilies based on their domain architecture: P and PPR-like (PLS), which are distinguished by motifs with no space and motifs with interspaced PPR-like motifs respectively. The PLS subfamily can be subdivided into five subgroups based on domain assembly at the C-terminus of a PPR protein: PLS, E1, E2, E+, and DYW (Figure 1) (Cheng et al., 2016; Xing et al., 2018). PPR proteins have been found in a variety of terrestrial plants since their discovery in yeast (*Saccharomyces cerevisiae* L.) (Manthey and McEwen, 1995). To date, PPR proteins have been found in many different plants, including *Arabidopsis* (Lurin et al., 2004), foxtail millet (Xing et al., 2018), poplar (Liu et al., 2016), maize (Chen L. et al., 2018), and rice (Chen G. et al., 2018), containing 441, 486, 626, 491, and 477 members of the PPR family, respectively. Additionally, PPR proteins have been discovered to have RNA-binding characteristics, allowing them to mediate gene expression via posttranscriptional mechanisms involving transcripts in the mitochondria, chloroplast, and nucleus. PPR proteins play vital roles in the plant organelle RNA editing machinery. PPRs could not only act as site recognition factors but also bind to *cis*-elements specifically. The resultant PPR–RNA complex and other editing factors, such as ORRM proteins and MORF proteins, can form a higher ordered editosome. As a result of their participation in different posttranscriptional processes, such as RNA editing (Hayes et al., 2015), RNA splicing (Ichinose et al., 2012), and RNA processing (Hao et al., 2019), PPR proteins are considered to have a substantial influence on organelle stability, including biogenesis and function. Furthermore, plant growth and development have been linked to the activities of PPR proteins. Previously, we briefly reviewed RNA editing in plant organelles, including the factors and mechanism of RNA editing, the editing events identified through deep sequencing data, and the roles of RNA editing (Ichinose et al., 2012). In this review, we emphasize on the recent discoveries of PPR proteins, including their roles in manipulating CMS-related genes, chloroplast biogenesis, embryogenesis, and stress responses. Furthermore, we also discuss the roles of PPR proteins in fruit growth, ripening, plant flesh colour, and fibre development.

The number of motifs in each protein can vary from 2 to 35, and the first motif can be any of P, P1, L1, S1 or SS. The E+ subgroup consists of proteins with a degenerate or truncated DYW domain.

### PPR Proteins Manipulate Cytoplasmic Male Sterility-Related Genes

Plant cytoplasmic male sterility (CMS) is a maternally inherited trait that maintains female fertility but results in abortive pollen. At present, CMS is considered to be jointly controlled by mitochondrial genes and their corresponding nuclear restorer (RF) genes, which can change the expression of CMS-related genes in mitochondria. Most *Rf* genes belong to the PPR gene family with several exceptions. Radish Rfo is a PPR protein containing 16 motifs. Inhibition of the *Rfo* translation process by binding to an *orf125*-transcribed mRNA inhibits the accumulation of the CMS-inducible protein ORF125 and restores CMS fertility (Koizuka et al., 2003). In addition, it is worth noting that sorghum *Rf1* encodes PPR13, which belongs to the PLS-E class and can restore male fertility by recruiting RNA editing enzymes (Klein et al., 2005). *OsRF1A* encodes pentatricopeptide repeat proteins, plays an additional role in promoting the editing of *atp6* mRNAs, and restores cytoplasmic male sterility in rice (Wang et al., 2006). Moreover, the PPR protein OsRF5 forms a complex with GRP162 to process *atp6*-*orf79* and restore fertility in the Honglian CMS line (Hu et al., 2012). Similarly, the interaction between the PPR proteins OsRF6 and Oshxk6 for coprocessing *atp6*-*orf79* rescues the male fertility of Honglian CMS lines (Huang et al., 2015). PPR756, a member of the PLS-E subclass, participates in RNA editing events of *atp6*, *ccmC*, and *nad7*. The loss of PPR756 could cause abortive pollen development in rice (Zhang Q. et al., 2020). The P-subfamily PPR protein OsPPR939, which can be phosphorylated by OsS6K1, regulates plant growth and pollen development by splicing mitochondrial *nad5* introns 1, 2, and 3 (Zheng et al., 2021). PPS1 is a mitochondria-localized PPR protein, while OsPGLl is a PPR protein that is localized to both mitochondria and chloroplasts. PPS1 is involved in 5 consecutive editing sites of the *nad3* transcript in mitochondria, while OsPGL1 is involved in RNA editing at a single site in both chloroplast and mitochondrial transcripts. The function of these two PPR proteins results in a decrease in the RNA editing efficiency at the specific site and ultimately in a defective phenotype concerning reproductive growth in rice.

### PPRs Are Required for Chloroplast Biogenesis

Previous studies have reported that 21 PPR and PPR-related proteins are required for plastid RNA editing in thale cress (Lu, 2018). Two PPR proteins (OsPPR4 and OsPPR6) have been shown to participate in the editing of a single plastid RNA editing site in rice; they are also involved in the editing of chloroplast RNA and are required for chloroplast biogenesis (Asano et al., 2013; Tang et al., 2017). The PPR protein AtPDM2 is located in plastids and regulates the expression of plastid genes related to chloroplast development by interacting with the organelle RNA editing factors morf2 and morf9. T-DNA insertion of the *AtPDM2* leads to the loss of pigment in *Arabidopsis* cotyledons and cotyledon albinism (Du et al., 2017). Another PPR protein in *Arabidopsis*, Hcf152 is located in chloroplasts and regulates the metabolism of chloroplast RNA by combining with the *petb* operon. This gene mutation will affect the accumulation of the cytochrome b6f complex (Meierhoff et al., 2003); A PPR gene in Poaceae, *Clb19* is required for editing the chloroplast transcripts *rpoA* and *rpoB*. Mutation of this gene will lead to impaired chloroplast development, a yellowing phenotype of seedlings and even death (Hein and Knoop, 2018). OsPPR16, a PLS-DYW subfamily PPR protein, is responsible for RNA editing of the RNA polymerase subunit RpoB and affects Chl synthesis and efficient chloroplast development in rice (Huang et al., 2020). The PPR protein DUA1 interacts with sigma factor 1 to form the PPR-SIG complex, and the module regulates chloroplast gene expression and chloroplast development in response to light and temperature (Du et al., 2021). The PPR protein OsPGL1 disrupts chloroplast RNA editing of *ndhD-878*, which is involved in the conversion of serine to leucine. Loss of OsPGL1 leads to the dysfunction of chloroplasts and the photosynthetic complex (Xiao et al., 2018). The PPR-SMR protein ATP4 participates in C-to-U editing of *rps8* RNA in rice and maize, which is required for the formation of photosynthetic complexes (Zhang J. et al., 2020).

### PPRs Regulate Embryogenesis

Previous studies have demonstrated that PPR proteins are essential for *Arabidopsis* and maize kernel formation, with the loss of function of specific PPR proteins resulting in empty pericarps and tiny, malformed kernels in various genetic backgrounds. AtEmb175 is located in chloroplasts and is the first PPR gene found in *Arabidopsis* that is related to early embryo death. Its mutation will lead to continuous cell division, embryo expansion, and abnormal tissue formation (Cushing et al., 2005). *ZmSmk1* encodes a protein containing a PPR structural domain that regulates seed embryo and endosperm development by regulating RNA editing of the mitochondrial gene *nad7* in maize (Li et al., 2014). The maize PPR-like protein EMP9 (EMPTY PERICARP9) regulates seed development by regulating RNA editing of the mitochondrial genes *ccmB* and *rps4* (Yang et al., 2017). ZmEMP21, a PPR-DYW protein that is needed for the editing of 81 mitochondrial target sites, is required for mitochondrial complex assembly as well as embryo and endosperm development (Wang et al., 2019). ZmPPR-SMR1 interacts with ZmCSF1 and is essential for the splicing of numerous group II introns, mitochondrial functions, embryogenesis, and endosperm development (Chen et al., 2019). The E-subgroup PPR protein DEK55 affects mitochondrial RNA processing, which is important for maize kernel formation (Ren et al., 2020). ZmPPR27 interacts with ZmMORF1 (MULTIPLE ORGANELLAR RNA EDITING FACTOR 1), regulates the RNA editing rate of mitochondrial genes such as *ccmFN* and affects the formation of the key mitochondrial protein complex, thus hindering seed embryo and endosperm development (Liu et al., 2020). The maize *DEK46* (DEFECTIVE KERNEL 46) gene encodes a protein containing a PPR domain that edits a specific site in the intron of the mitochondrial *nad7* gene. When DEK46 is functionally absent, the percentage of selective splicing of the intron of the mitochondrial *nad7* gene is reduced, which in turn affects normal seed development in maize (Xu et al., 2020). A DYW domain–containing PPR protein, PPR2263, was found to be involved in RNA editing in the mitochondrial NADH dehydrogenase 5 (*nad5*) and cytochrome b (*cob*) in maize. The *ppr2263* mutant showed reduced embryo and endosperm growth, resulting in growth defects in kernels and seedlings (Sosso et al., 2012). A PPR protein, defective kernel 2 (Dek2), is required for *nad1* mRNA splicing in maize. The *dek2* mutant displayed small kernel and tardy development. (Qi et al., 2017). Another E+ subgroup PPR protein, DEK40, involved in the processing of cox3, nad2, and nad5 was identified to be essential for mitochondrial function and kernel development in maize (Ren et al., 2019).

### PPRs Participate in Stress Responses

RNA editing may have contributed to the adaptation of land plants to extreme temperature, UV, and oxidative stress during the early stages of land plant formation (Fujii and Small, 2011). Increasing molecular evidence has revealed that many PPRs are involved in the response to a variety of biotic and abiotic stresses. In *Arabidopsis*, salt, oxidative, and ABA stressors all increased the expression of the *PPR96* gene (Oren et al., 2001). The PPR protein GUN1 is associated with plastid-to-nucleus retrograde communication, control of *ABI4* expression, and photooxidative stress responses in *Arabidopsis* (Koussevitzky et al., 2007). In *Arabidopsis*, PPR40 is known to offer a signaling connection between mitochondrial electron transport elements. PPR40 knockout resulted in increased reactive oxygen species (ROS) accumulation, lipid peroxidation, and superoxide dismutase activity (Zsigmond et al., 2008). The PPR protein ABA overly sensitive 5 (ABO5/At1g51965) is required for NADH dehydrogenase subunit 2 (NAD2) intron 3 splicing in mitochondria. Compared to the wild type, the *abo5* mutant accumulated more H_2_O_2_ in roots (Liu et al., 2010). MITOCHONDRIAL RNA EDITING FACTOR 11 (MEF11)/LOVASTATIN INSENSITIVE 1 (LOI1) controls isoprenoid production, which is known to influence defense gene expression in response to wounding and pathogen infection (Kobayashi et al., 2007; Tang et al., 2010).

The *Arabidopsis* PPR-like protein AHG11 (ABA HYPERSENSITIVE GERMINATION 11) can edit the mRNA of the mitochondrial gene *nad4* to maintain normal intracellular levels of reactive oxygen species (Murayama et al., 2012). PGN (PENTATRICOPEPTIDE REPEAT PROTEIN FOR GERMINATION ON NACl) has been shown to be involved in biotic and abiotic stress responses (Laluk et al., 2011). Functional disruption of the PPR protein SLG1 impacts mitochondrial RNA editing, plant growth, and abiotic stress responses in *Arabidopsis* (Yuan and Liu, 2012).

Plant development is also regulated by another PPR protein, SLO2. Stress-sensitive genes have higher transcript levels in the *slo2* mutant. Furthermore, the *slo2* mutant is hypersensitive to osmotic and ABA stressors at various phases of seed germination, although their mature plants have a high resistance to salt and drought stresses (Zhu et al., 2012; Zhu et al., 2014). SVR7 (SUPPRESSOR OF VARIATION 7) is needed for chloroplast ATP synthase subunit translation in *Arabidopsis*. SVR7 knockout led to increased ROS production, increased sensitivity to H_2_O_2,_ and decreased photosynthetic activity (Lv et al., 2014). SOAR1 (suppressor of ABAR-overexpressor 1), which encodes a nucleocytoplasmic localized PPR protein, has recently been discovered to be a positive regulator of the responses to different stresses, including those to drought, salt, and cold (Jiang et al., 2015).

### PPRs Regulate Fruit Growth, Ripening, Plant Flesh Colour, and Fibre Development

With the discovery of growing molecular evidence, researchers have found that PPR genes are involved in a variety of fruit functions, including as growth, ripening, colouration, and fiber formation. *GUN1*, which encodes a plastid-localized PPR protein, has been involved in the plastid-to-nucleus retrograde signalling route during tomato fruit development and ripening (Pesaresi et al., 2014). A number of tomato mutants, such as *Cnr*, were found to have dramatically reduced the expression of ripening-related PPR genes, resulting in mature fruits with colorless pericarp tissue, indicating that PPR proteins play a role in the development of fruits (Eriksson et al., 2004). *CmPPR1*, which encodes a plastid-targeted P-type PPR protein in melon (*Cucumis melo* L.), has been identified as a potential main quantitative trait locus (QTL) that determines flesh colour intensity (Galpaz et al., 2018). It was shown that genotyping 70 lines utilizing four SNPs from four *ClaPPRs* resulted in match rates above 0.87 for each validated SNP in association with the distinct phenotypes of skin colour. These findings contribute to a better knowledge of PPR genes and their functions in watermelon fruit growth and ripening, which may be useful in watermelon cultivar improvement (Subburaj et al., 2020). *GhImA*, which encodes a PPR protein, is involved in mitochondrial *nad7* splicing, respiratory metabolism, and cotton fibre formation via ATP supply and ROS balancing (Zhang et al., 2021).

## Conclusion and Perspectives

In general, PPR proteins participate in plant growth and development by RNA editing or RNA stabilization and splicing. Recently, many attempts have been made to engineer special proteins for efficient RNA editing in plant organelles. Shen et al. observed the interactions between different PPR codes and RNA bases at the atomic level, revealing the molecular basis for the modular and specific recognition patterns of the RNA bases U, C, A, and G (Zhang et al., 2021). PPR family proteins have the ability to enter organelles and bind to single-stranded RNA, and more recognition codes are being studied and verified. On this basis, according to the single-stranded sequence characteristics of target RNA, the corresponding PPR proteins have been artificially designed. Developing RNA-targeting tools will likely accelerate the functional study of RNA editing and the biological study of chloroplasts and mitochondria. By fusing the corresponding functional domains, the artificial design of fused PPR proteins is expected to become the next generation of biotechnology that can regulate the expression of organelle genes.
